# Contextualizing post day-one childhood immunization in-take drop-off rate in Nigeria: An assessment of working mothers in Ibadan

**DOI:** 10.12688/gatesopenres.15135.2

**Published:** 2025-05-27

**Authors:** Mofeyisara O. Omobowale, Folakemi A. Amodu, Olugbenga S. Falase, Taiwo H. Olajide, Olukemi K. Amodu

**Affiliations:** 1 Institute of Child Health, University of Ibadan, Ibadan, Oyo, Nigeria; 2 Department of Sociology, Lead City University, Ibadan, Oyo, Nigeria

**Keywords:** Post-day one immunization, Working mothers, Drop-off rate, Ibadan

## Abstract

**Background:**

Contextualizing childhood immunization in the context of children of working mothers can boost coverage and completion interventions. This study examines how informal working mothers perceive post-day-one routine immunization, and vaccines not covered under the National Program on Immunization (NPI), immunization schedules, timing, and duration.

**Methods:**

The study utilized a mixed methods approach, including in-depth interviews and semi-structured questionnaire to capture immunization experiences and assess the context of post-day one. The study was conducted in Ibadan, Nigeria and involved 1,044 quantitative and 73 qualitative samples of working nursing mothers. Data were analyzed using descriptive statistics, chi-square test for proportions, and t-test for means (p<0.05), while qualitative data were subjected to content and thematic analysis.

**Results:**

The average age of mothers participating in this study was 31.39±6.52 years. The mean age of children of mothers recruited into this study is 19.26 ±16.14 months. Majority of these mothers (95%) are married. Around three-quarters of women in this population ensured immediate immunization for their infants after birth, but less than a third achieved the complete age-specific vaccination series due to livelihood related causes, long waiting time spent in conventional immunization clinic. Around 40% of interviewed mothers vaccinated their children up to the third DPT dose, and just over 30% achieved full vaccination. Many informal working mothers, have practice of adding 'supplements' to their children's immunization, driven by a lack of sufficient information about the vaccines. Some mothers also seemed unaware of these specialized vaccines.

**Conclusions:**

Promoting complete immunization requires more than just raising awareness about childhood vaccinations but close and quick immunization service delivery is required. It is crucial for mothers to possess comprehensive knowledge about the mechanics and operation of immunization. Achieving this understanding could involve translating vaccine names and functions into indigenous terms, enhancing clarity and comprehension. Furthermore, a firm grasp of the immunization schedule significantly contributes to successful immunization completion.

## Introduction

Childhood immunization has become an important contributor to the control and management of mortality and vaccine-preventable diseases among children in low-income countries ( Abbas *et al.*, 2020; Hilton *et al.*, 2006). Nigeria has developed several strategies for combating vaccine-preventable diseases since 1979 when it adopted the expanded program for immunization (EPI), and these efforts have drastically reduced the rate of under-five morbidity and mortality. This is further evidenced by the country recently attaining a polio-free status ( Ekwebelem *et al.*, 2021). However, a low completion rate of immunization, low coverage and increased drop-off rate are among major challenges militating against arrays of approaches, which low-income countries like Nigeria have deployed to increase completion, coverage and demand ( Adedokun *et al.*, 2017).

Nigeria is still plagued with one of the highest rates of under-five mortality in the world ( Matthew Ayodele *et al.*, 2024; Mutiu *et al.*, 2019), the second largest after Pakistan ( Asim *et al.*, 2015). Nigerian 2016 Multiple Indicators Cluster Survey/National Immunization Survey Coverage (MICS/NICS) revealed that 77% of children aged 12–23 months in Nigeria have not received all the routine vaccinations according to the recommendation of the national EPI schedule. In addition, 40% of children in the above age group did not receive any vaccinations, meaning that the 90% national target that has been set by the country was not met. Likewise, 33% of children aged 12 to 23 months received three doses of Penta vaccines, while 31% of children who received Penta 1 vaccines did not complete the three-dose series ( Oleribe *et al.*, 2017; WHO, 2017). Overall, the national immunization coverage average is still far from the World Health Organization (WHO) recommended coverage rate ( Chido-Amajuoyi *et al.*, 2018; Obiajunwa & Olaogun, 2013; Oleribe *et al.*, 2017). The 2018 NDHS reports show that the percentage of children aged 12–23 months who have received all basic vaccinations was 43.0% in south West Nigeria, while only 34.9% of this age group have received all age-appropriate vaccinations in the region. Individual states have varying levels of vaccine uptake such as Oyo state in the South-Western region with 23.3% coverage in the number of children of all basic vaccinations and 16.6% with all age-appropriate vaccinations. The average immunization coverage of the state is low and puts the region behind the South-South and South-Eastern regions of the country with higher coverage ( McGavin *et al.*, 2018).

Studies over the years, have revealed many contextual determinants including but not limited to mother’s educational status, employment status, income, age, marital position, religious inclination, ethnic division, child’s age, birth order and ease of accessing health center to influence the uptake and completion of childhood immunization ( Adenike *et al.*, 2017; Mutiu *et al.*, 2019; Omobowale, 2021; Ophori *et al.*, 2014; Tagbo *et al.*, 2012). Similar studies conducted in different locations and populations in the country revealed a largely similar set of factors militating against the uptake and completion of immunization by children all over Nigeria with the factors being stronger determinants among some populations as compared to others ( Abdullahi, 2018; Adeloye *et al.*, 2017; Adeyinka, 2012). The variation in the nature and impact of these factors in different sub-cultures/population of the country is an indication of the contextual differences in population subsets, which have been limitedly explored. Contextualizing post-day-one childhood immunization among working mothers as a subset of Ibadan, cultural group encompasses examination of interpretations, understanding within a trick of social/cultural perspectives, the nature of social/cultural reality, social explanations of knowledge, and interactions regarding childhood immunization as a social (health) process in childcare.

Maternal factors and the context in which they manifest play an important role in the access, uptake, and completion of immunization of under-five children. These contextual maternal factors consequentially affects the immunization coverage rate in the country ( Fatiregun & Okoro, 2012). Statistically, children of young mothers (15–24 years), illiterate mothers, mothers who did not attend ante-natal clinics, mothers who delivered at home or maternity homes, mothers who had no access to media, and mothers who had little, or no knowledge of immunization were more likely not to complete or receive any immunization ( Adedokun *et al.*, 2017; The National Demographic Health Survey, 2013). Children of mothers who are aware of immunization at birth are 1.9 times more likely to be fully immunized, while children of mothers who had secondary and tertiary education are two times more likely to be fully immunized than those children whose mothers had primary or no formal education ( NDHS, 2018). Other important factors that are maternal-related are the level of education, higher income, and easy access to healthcare establishments among others ( Fatiregun & Okoro, 2012). Although, several studies have explored factors that are associated with the low vaccine uptake among Nigerian children, which have been attributed to low immunization coverage, nursing mother’s poor knowledge about immunization, and educational status, among other factors ( Abdullahi, 2018; Abdulraheem *et al.*, 2011; Antai, 2010; Antai, 2012; Anyene, 2014; Oladepo *et al.*, 2019). The contextual understanding, the interplay between social/cultural interpretations, explanations and interactions that dictates the social reality of working in the informal sector and the up-take of childhood immunization beyond first schedule and post day-one in South-Western Nigeria, needs to be explored.

The success of completing the immunization exercise is contingent on the contextual experiences and understanding of mothers who value the significance of childhood immunization. Contextualizing childhood immunization will advance appropriate intervention that will help in increasing immunization coverage and completion among working mothers. The proper monitoring of children in the immunization routine activities cannot be detached from the socio-cultural informed understanding of mothers who are the primary caregivers. This study explored the contextual understanding of informal working mothers on post-day-one childhood immunization in different specific areas including the contextual understanding of the concept of immunization, vaccines not under the National Program on Immunization (NPI), immunization schedule and period as well as the timing and duration of the immunization.

## Methods

### Ethical considerations

Ethical approval was received from both University of Ibadan/UCH Research Ethics Committee (UI/EC/20/0058) on 23/05/2020 and Oyo State Research and Ethics Review Committee (AD 13/479/1777B) on 20/05/2020. During the data collection, the study details, purpose, and participants’ right to privacy were explained to all participants, with the clarification of the right to withdraw at any time from the interview. Informed consents were obtained both verbally and written. The participants in this study were not exposed to any serious know risk. The research team employed various means of protection to minimize risk. Their participation in this study cost them nothing invasive, incentives and transportation were provided at every required instant. Participants through the findings and discussions of this study were more enlightened about childhood immunization along with other minor health-related services benefits provided to them.

### Study description

A mixed methods approach was adopted for this study to gain a more comprehensive knowledge of the phenomenon studied. The study was conducted in Ibadan, a major city in Oyo state. The city hosts large markets with a huge population of mothers from diverse cultures in Nigeria. A total of 1,044 (quantitative samples) and 73 (qualitative samples) nursing mothers were sampled from both rural and urban markets of Ibadan, using explanatory sequential mixed methods design. A pretested interviewer administered questionnaire, immunization record assessment, and in-depth interviews with mothers and health workers were employed in the study. The eligible study population comprised of all consenting nursing mothers working within the markets who have commenced childhood vaccination, and health workers in the selected study sites. A total number of 1,044 working mothers were sampled from 13 randomly selected markets (Agbeni, Bodija, Gbagi, Oje, Oja Oba, Ojoo, Dugbe, Sango, Mokola, Orita Merin, Bode, Olomi, Ikereku, Olulosin, Ogunranti 2 and Academy) in Ibadan metropolis, Nigeria for the study.

The qualitative data were obtained through unstructured interview guide for in-depth interviews (IDI) with 73 consenting purposively selected working nursing mothers in selected markets. Four IDIs were conducted in all markets except in Bodija, Agbeni and Gbagi markets where 7 IDIs were conducted due to the larger number of nursing mothers in the markets. The interviews were stopped when saturation was reached. In-depth interviews help deepen knowledge by bringing focused, insightful and improved understanding of the study ( Brounéus, 2011). The questionnaire were written in English and Yoruba Languages, running it through processes of translation and back translations by experts in the language. Mothers of children below the age of five years in selected markets were, purposively identified, approached for explanations and consent for participation in the study. Non-consenting mothers were exempted, while all consenting mothers either verbally or written were recruited for the study. However, in all markets, market leaders and significant others of the mothers had earlier been identified, visited, and carried along from the inception of the study. The in-depth interview focused on nursing informal mothers with children below 5 years of age to sufficiently explore, understand, and contextualize post-day-one childhood immunization narratives, challenges, and possible solutions. Interviews were transcribed, and coded using process coding methods when observable and conceptual action in the data were linked to process codes, intertwined with the dynamics of vaccination time, such as things that emerge, change, occur in particular sequences, or become strategically implemented. Process coding is appropriate for virtually all qualitative studies, particularly for grounded theory research that extracts participant action/interaction and consequences ( Miles & Huberman, 1994). The data were also categorized and subjected to content analysis.

### Statistical analysis

The quantitative dataset was entered and analyzed using statistical packages for Social Sciences (SPSS, version 20 windows). Descriptive statistics of the demographic details of the recruited mothers were done. Results were represented by numbers, percentages and expressed by mean. Chi-square test was used to observe the difference between the proportions, t-test was applied to observe- the difference between the two means for normally distributed continuous data. A p-value less than 0.05 was considered as significant.

## Results

### Demographic information of working mothers in Ibadan

The mean age of mothers enrolled in this study was 31.39 ±6.52 years and the average age at first parturition is 24.12±4.38 years. The mean age of children of mothers recruited into this study is 19.26 ±16.14 months. The mean number of days from birth to first vaccination for children of mothers in this study was 1.95 days and the average number of children per mother in our study is 2.43. Half of the mothers in this study (53.8%) completed secondary school. About one-fifth of the study population had tertiary-level education, while more than a third of the population had no form of education. The distribution of educational levels of mothers is presented in Table 1. The majority of the mothers (95%) in this study were married while all the others are single mothers including never married, divorced and widowed. Most mothers (82.9%) that had antenatal care during their pregnancy attended health care facilities for their antenatal services, while others patronized unorthodox care centers for their antenatal care, including 13.6% that had their antenatal care in faith homes. About 76% and 16.4% of respondents in this population delivered their last child at a healthcare facility and mission house, respectively while others delivered their babies at other unorthodox care centers (see supplementary figure). About three-quarters of the women in this population immunized their children immediately after birth. Less than a third of the children in this population had completed the age-specific vaccinations. (see supplemental tables)

**Table 1.** Educational attainment of working mothers.

Highest level of education			
Educational attainment	Frequency	Percent	Cumulative Percent
No education	37	3.7	3.8
Others	4	.4	4.2
Quranic school uncompleted	2	.2	4.4
Quranic school completed	1	.1	4.5
Primary uncompleted	4	.4	4.9
Primary completed	72	7.2	12.1
Secondary uncompleted	111	11.1	23.3
Secondary completed	536	53.8	77.0
Tertiary uncompleted	49	4.9	81.9
Tertiary completed	180	18.1	100.0
Total	996	100.0	

*Table showing the highest educational attainment by mothers in the study. Note that 48 mothers do not disclose their educational status.*

**Figure 1.**
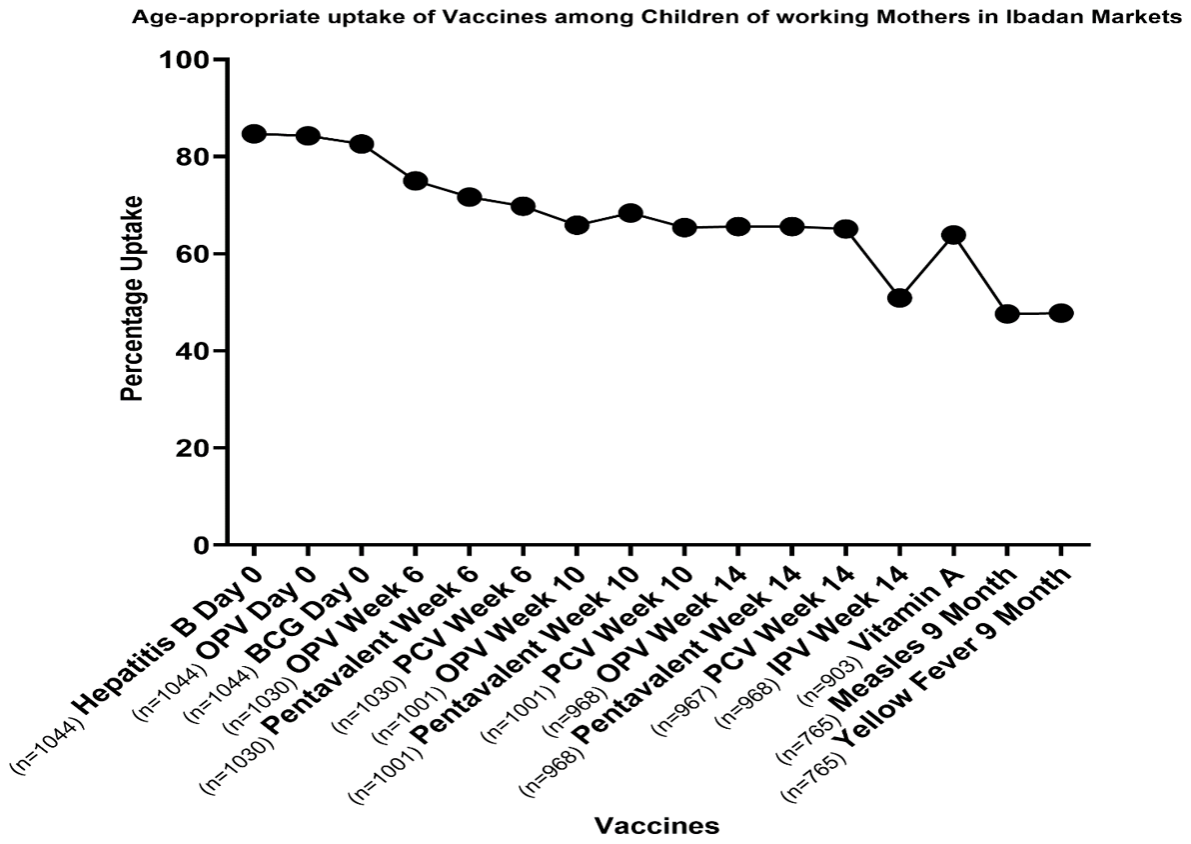
Age Specific completion rate of each vaccine among children of informal working mothers in Ibadan Nigeria. The Figure 1 shows the progressive decline in the number of children taking their vaccinations as they advance in age. The most vaccine taken in the population is the first dose of Hepatitis B and the lowest taken vaccine is the yellow fever vaccination taken at age 9 months ^1^. The dropout rate compares the number of infants who begin the immunization schedule to the number who complete it. And the completion rate here refers to fully vaccinated children, those that received the complete recommended vaccination that is appropriate for age as recommended in the National Immunization scheme.

More than half of the women vaccinated their children on day zero, while about 22.4% of mothers vaccinated their children within the first week of birth ( Table 2). Mothers in the urban markets are more likely (OR-1.934 C.I 1.317-2.841) to have completed the age-specific immunization schedule for their children (14th week -42.57% ,9th month -33.95%) when compared to their counterparts in the rural market. More than half (53.3%) of the children of mothers in the rural markets dropped off the immunization schedule before they were fully vaccinated. As presented in Figure 1 below, almost 40% of the mothers interviewed vaccinated their children till the third dose of DPT and just a little above 30% completely vaccinated their children. There was a steady decline in vaccine uptake with the increasing age among children in the Ibadan population as depicted in Figure 1 below.

**Table 2.** Age at which children of working mothers started immunization after birth.

Age	Frequency	Percentage
Day 0	554	53.1
Days 1-7	234	22.4
Days 8-14	43	4.1
Day 14 above	213	20.4
Total	1044	100.0

*The table depicts the distribution of the timing when newborns received that first vaccination in the population. About half of the population were not vaccinated at day zero and as much as one-fifth of the population got their first vaccine at above two weeks of age.*

**Table 3.** Childhood immunization completion rate and drop-off rate among working mothers by Market region stratification.

Population Stratification		Summary Statistics			
		Completion at 14 weeks	Completion at 9 Months	Drop off rate	Total Population
Market Population	Urban	367(42.57%)	293(33.95%)	358(41.55%)	863
	Rural	48(26.80%)	38(20.99%)	97(53.3%)	181
	Total Study Population	415(39.81%)	331(31.7%)	455(43.6%)	1044

*Table showing the age-specific immunization completion and drop-off rates in the population.*

### Informal working mother’s contextual understanding of childhood immunization

To clarify the understanding of the informal working mothers on childhood immunization, various questions from the perspectives of working mothers were sought, which included their understanding and interpretations of the reason for childhood immunization. One of the informal working mothers defined immunization as a disease-preventing vaccine to secure the future as thus:

Immunization is those vaccines that we take to prevent diseases that can affect children in the future. It is something like preventing. We take Hepatitis B, Diabetes, Hepatitis B1, and B2, we just collected it (Agbeni Market IDI).

The mother here has a layman understanding of vaccines as prevention but seems not to know some of the vaccines and their functions as she added diabetes to the list of mentioned vaccines. Another working mother whose opinion was generally shared by many mothers explained the importance of childhood immunization from a preventive perspective also:

I know that it prevents paralysis and it is good for a child. It also prevents *jedo-jedo* (Hepatitis) and diseases like *romolapa*- *romolese* (Polio) and *yinrun*- *yinrun* (Meningitis) (Agbeni Market IDI).

Post-day-one childhood immunization is an exercise that should be taken seriously. In explaining some of the diseases that post-day-one immunization prevents, a working mother emphasized that a good mother needs to take immunization very seriously by ensuring that all children take it. She said:

Immunization is good and a true mother should make the effort to ensure that she takes it for her children completely, and we can see its work in our children. So, it is very good and necessary (Agbeni Market IDI).

Confirming the above statement, a mother also opined that:

I know that immunization is important for a child and it is something good that parents should take for infants. It prevents diseases such as polio, diarrhea, tuberculosis, and measles (Gbagi Market IDI).

A respondent emphasized the reason and importance of childhood immunization and also as a means of preserving culture and socialization processes because the immunization exercise is a continuous process that is passed from one generation to another:

I know that it’s good because the parents who gave birth to us also immunized us when we were young. So, when we give birth to a child, we need to immunize them also (Gbagi Market IDI).

Obviously, it can be deduced from the narratives above that the informal working mothers have the layman's knowledge of childhood immunization. Most informal working mothers see childhood immunization as an exercise to prevent children from diseases and other ailments, which may affect children. Also, immunization is recognized as a means of preventing children from sudden death and securing their future.

However, in spite of the working mothers’ awareness on childhood immunization, some of the mothers do not know the specific vaccines their children receive. Many of them take the vaccines as instructed and on the general assumption that ‘it is good for children’ as adduced by one of the interviewees:

We have been taking it (immunization), we have taken all the ones that the child is supposed to take, and the ones they carry around that they bring to the market, we also take that one. But I don’t know the name of the vaccines, they only said it is good (Agbeni Market IDI).

As a result of inadequate information that exists on the kind of immunization that children receive, it was observed and confirmed that some working mothers use ‘pharmaceutical supplements’ as an addition, a practice, which could be detrimental to the health of the children. A mother emphasized thus:

I don’t know the name of the vaccine when it is not the only one. We also use *Babyrex* and vitamins for her [the child], and when we go to the clinic, they say that one is for *romolapa-romolese* (polio) or something like that. Also, I used to hear the advertisement, because they don’t write the name of the vaccine for us, they just write 3 letters, and you know it is only the nurses who know what they mean by that (Agbeni Market IDI).

Yet another working mother noted that:

I don't know the names, but they say some prevent measles, some for polio, yellow fever, and so on. So, all I know is that they prevent those diseases (Bodija Market IDI).

Although the immunization card has a record of the vaccines received and the next vaccine to be administered, most mothers do not understand the actual vaccine to be received as explained by a working mother whose child had incomplete vaccination thus:

It (Immunization) is good, it is very good for the body. It kills all diseases that are in the body, such as fever, cough, *iko ahubi* (coarse cough), and *iko ahugbe* (dry cough). It is good for children, the way they give them in stages is how we take it, and we have the record. They gave us cards. When we were supposed to receive it, we went to the place and they gave us the next date and wrote the next vaccines that we would take on the next appointment. (Bodija IDI Market).

Informal working mothers, in most cases, do not also know and understand the specific vaccines given to their children. While they are aware of when their children were given the previous immunization through the immunization card, in most cases, they are oblivious of the specific vaccine received as well as the functions but rely on the health workers to tell them. A working mother opined that:

When we gave birth after 7 days, they gave the baby at the hand, and buttocks, after that, at the two hands when she was 2 months but I can’t remember the name of the vaccine. It is good to take it. For the 3 months, I don’t know the name of the vaccine. When we get there, the nurses will explain it to us (Bodija Market IDI).

Likewise, another working mother said:

I can’t remember the vaccine that was given to my baby but it is in my baby’s card. They usually write the one that he will receive there, but I can’t really remember. I know BCG and Rotavirus, the one that prevents meningitis, but I don’t know the timing of these vaccines, despite the fact that I have four children (Gbagi Market IDI).

The above indicate lack of informed understanding of vaccine among mothers may have implication for of timely and complete vaccination of their children across groups.

### Informal working mother’s understanding of vaccines not under NPI

Most informal working mothers are informed about ‘special vaccines’, which are not under the NPI, these are government-approved but not subsidized vaccines. They know that these vaccines exist and are good for children, but the vaccines are not free. There are suggestions that all working mothers should get these vaccines, but, the cost of these vaccines, remains a major problem for most mothers as explained by one of them:

Of all the vaccines, the BCG is very important, the PCV, and the Rotavirus. They are all good. For the Rotavirus, if not that it is expensive for most of the parents, it would have been good for everybody to take it for their children (Gbagi IDI).

A working mother who has been informed but could not afford one of the special vaccines opined that although the vaccines are crucial for the children, the government needs to support parents by subsidizing the cost of the special vaccines.

Yes, there is one that they said is ROTA (Rotavirus), but it is too expensive, and I feel if it is something that is very important the government will subsidize it and it will be free for everybody so I am not bothered about it but some people still go to pay for it and take for their child. Out of a hundred maybe few people will be able to afford that money. So, I did not bother to immunize my child against Rotavirus. I only take the free ones. It is only Rota that I know is not free (Agbeni Market IDI).

An informal working mother who knows more about the functions of the special vaccines talked about the time that a child should get them. She explained that:

So, the vaccines that we are talking about, are what we call special vaccines. The one that stops *Igbegburu* (Dysentery), there is Meningitis, and different ones are available. Those ones, would not tell you at the clinic that they are available. Like the one for Rotavirus, a child between 6 weeks and 10 weeks, is supposed to receive it. So, some are to be taken after one year, one for 9 years for females (Agbeni Market IDI).

However, some working mothers do not know if there are special vaccines that require any payment. While responding to a question on the special vaccines, a woman surprisingly said:

Is that also an injection [vaccine]? We don’t pay for any vaccine in the place where we take vaccine o, we don’t pay for the vaccine (Agbeni Markets IDI).

Correspondingly, a working woman who is completely unaware of special vaccines also explained that:

They should let us know about it [special vaccines], because I have never heard of it. Even the first one we received was at the local government, the one for three months or six weeks, and after birth before naming. It was at a local government that we received it (Agbeni Market IDI).

However, a woman was able to get one of the special vaccines for her children in order to prevent diseases.

I have heard one that prevents meningitis. It is a good vaccine because I got it for my 2nd and 1st child, because of the disease in the area. We are told to get it for them after 1year (Agbeni Market IDI).

The quantitative data of this study also confirms that the completion rate for special vaccines, particularly, Rotavirus ^2^ is very low as only a 7.5% rate had been completed. Clearly, it can be inferred that the available special vaccines are not free, hence, they are unavailable in many immunization clinics and unaffordable to mothers who live barely on and/or below the poverty line ^3^. by the working mothers. What inhibits most mothers from getting their children vaccinated with these “special vaccines” was the associated cost of the vaccines. Besides, some working mothers do not know that these vaccines exist and they do not have the knowledge of their functions. For the vaccines to be accessed and affordable, the government needs to subsidize and made them easily accessible.

### Informal working mother’s understanding of immunization schedule and period

Childhood immunization schedules and period guide mothers on when to take their child(ren) for the next immunization appointment. There is no doubt that the immunization schedule and period will be taken seriously based on the contextual understanding of mothers. Even with the immunization card as a reminder, working mothers who are too busy with their daily work or who do not understand the importance of immunization are at risk of missing the next period of immunization. A mother explained how informed she was on the immunization schedule:

Yes, they tell us. When the child was 3 months, they tell us to come for vaccination, then for 6 months, and after 6 months. They [Nurses] tell when to come next whether by 8 months or 9 months and the date for the next appointment is written in our immunization cards so that when you look at it you remember when to go for the next appointment (Agbeni Market IDI).

While referring to the lecture received by health workers, a mother stated the specific times given to her on the immunization schedule as:

At birth, one month, 3 months, 6months, 9months, and 1 year, that was how we were lectured (Agbeni Market IDI).

Similarly, another woman explained the immunization schedule as starting from:

A day after birth, 41 days, 2 months, 3 months, 6 months, 9 months, and 1 year (Agbeni Market IDI).

In addition, an informal mother emphasized the immunization schedule with personal experience:

At birth, 2 months, 3 months, 6 months, although the vaccine is given in the child's mouth, 9 months, and 1 year. I know a child is supposed to complete the vaccination at 2 years because when I gave birth to my 2nd child, the schedule was completed at 18 months but for my 1st born, it was completed at 12 months so now I think the vaccination schedule ends at 2 years (Agbeni IDI).

However, a woman who knows that various vaccines exist for the immunization of children but does not know how and when to take them for her child responded:

There are a lot of vaccines like my baby now has taken the vaccines like six times. But I do not know how many vaccines a child should take (Bodija IDI).

Different from the unknown woman, another working mother makes use of any available moment to immunize her child as explained thus:

I take it from any clinic depending on my location at that moment and I sometimes take the child to the father's village, because the healthcare people often come there. Recently, when I came home, I was passing through Ojee, and I came across some set of healthcare persons giving vaccines, and I took my child to them (Agbeni market IDI).

Another woman emphasized that:

On the day I took the first one, if it was after a month or three months, they would have given me the date for my next appointment. So, it’s the date that I bear in mind to go next, so when I get there, if it’s available, fine I will take it, and if it’s not, they will tell me to come the following week (Gbagi market IDI).

However, the schedule and period of immunization make it easy for informal working mothers to know when and where to immunize their child(ren). It serves as a guide as one woman noted:

You know it is always scheduled, so that makes it easier. Just like after giving birth, you get some; there is another one at six weeks, then ten weeks, 14 weeks then, 9 months, 1 year, and the like. One will know how to do it (Agbeni market IDI).

A mother that understands the importance of the immunization period finally concludes that:

I usually don’t want to miss the dates. I make sure it is the exact date that I go for the immunization except if I was not chanced to go on that day. But, I don’t miss it at all, so that it [immunization] can be complete in the body of my children (Gbagi market IDI).

Many mothers do not seem to know danger associated with time and vaccine uptake schedule. It is taken like other conventional hospital appointments. Some mothers missed these appointments without rescheduling/or revisiting clinics until after many months. Some for the fear of been scolded by health workers for negligence others for lack of time due to heavy economic burden.

### Informal working mother’s understanding of the timing and duration of the immunization

On the duration of the vaccine, it was observed that there was no uniform period according to the responses of the informal working mothers. While some believe that the immunization period ranges from birth to 9 months, some mothers agreed to a year, yet some others said it is over a year, and even more. One of the women noted that:

For my firstborn, I stopped collecting immunization for him at one year and six months, but I am still collecting vitamin A, which they put in his mouth (Agbeni market IDI).

It was observed that some groups of working women agreed that immunization starts from birth till 9 months, while some women noted that the duration is between the delivery period and one year. A woman narrated her experience on the immunization period for her children thus:

When we were collecting the vaccine for the older ones, the vaccine used to stop at one year, but for the ones that I gave birth to later, it was one year and 6 months, now they say you can receive for 2 years (Bodija market IDI)

The uncertainty in the duration of the vaccine also persists as explained by a working mother who missed some vaccinations for one of her children earlier but hopes to complete it for others as she explained that:

Ehn, we did not know then, the one that is now 3 years old, if he can still it, I would take him there. The one that is 10 years old, if he can receive it, I would take him there too. There is one that is 8 years old too and one that is 2 years old now, I have said that I would take him to receive it (Bodija market IDI).

Children, at times, may miss the immunization period as a result of a lack of experience on the duration of the vaccine as an interviewee simply said:

Yes, my child was immunized, but it has been a long that he has been immunized (Bodija market IDI).

A woman who was unsure about the timing and duration of the immunization needs to rely on the information given by the health workers:

Hmm, they used to tell us a lot about it, but there is little I can remember. Although at birth he took one, I think vitamin K. Also, in the fourth month, he took another one, A month after, he took another one again till the sixth month. I think we took a vaccine that cost seven thousand naira in the third month. In the sixth month, he took vitamin A, which was dropped in his mouth to prevent what he will be eating. After that, we went there but they did not give him any vaccine they just asked questions about his body's reaction to food. We were told he would take another vaccine at nine months (Gbagi market IDI).

A woman narrated that the immunization process should be done “Immediately one gives birth to the baby like the second day after birth and getting home, one should go and receive it. In fact, my child had not been named before I started the immunization. I went to the one at nine months yesterday. So, I take it for my child. Then a year and they also said when the child is a year and three months, I should bring him back. They’ve written it on the card (Gbagi market IDI).

Mothers lay importance on receiving the first set of childhood immunizations.

## Discussion

The contextual understanding of informal working mothers plays a significant role in childhood immunization uptake in Ibadan. The working mothers in informal spaces represent a unique population that strives to balance multiple needs and tasks including livelihood survival and childcare practices simultaneously ( Oladokun *et al.*, 2009; Omobowale, 2021). Mother literacy and religious institution education on immunization are two important factors in achieving optimal childhood immunization in the study population. This corroborates the findings of Ijarotimi *et al.* (2018) on the immunization status of children and associated demographic factors in Akinyele Local government are of Oyo state, that female literacy and targeting religious institutions may be effective in improving immunization uptake. The majority of mothers in this study were traders, demonstrating that maternal livelihood may also prevent optimal uptake of immunization by children of working mothers in the informal sectors. Many working mothers in the informal space like markets are pressed with demands of sales and profit making in order to sustain their livelihood. In some cases, many of them despite being married, still remained “breadwinners” in their families ( Tade, 2022). Some of them are also indebted to serving usury loans especially that of *gbomu le lanta*. “Usury loans are locally described as *owo gb’omulelantan* (which literarily means “a loan akin to having one’s breasts on a hot lantern”). In short, a usury loan default worsens the livelihood and economic situation of the loanee” ( Omobowale *et al.*, 2020) and thus may affect their abilities to keep immunization schedules of their ( Oladokun *et al.*, 2010; Omobowale, 2019).

The report of Obiajunwa & Olaogun (2013) from an urban-rural study on immunization revealed an immunization completion rate of 26.5% (Urban), and 31.7% (Rural) of mothers in our population state their children have completed the age-specific immunization routine at the time of conducting this study. The reason for the marginal difference in the completion rate we reported here could be associated with higher education status and 100% employment status among our study population. We report a completion of 82.6% for BCG, 71.1% for DPT1, 68.4% for DPT2, and 65.7% for DPT3, which were markedly higher than the completion rates reported in a south-eastern population of Nigeria. The 84.3%,75%, 65.9%, 65.6%, and 47.6% completion rates for OPV0, OPV1, OPV2, OPV3, and Measles, respectively, were consistently lower than the reported among the same south-eastern population of Nigeria ( Fatiregun & Okoro, 2012). The differential completion rates among the vaccines between the comparison populations are indicative of a yet to be uncovered population-specific reason influencing the vaccine uptake rates in the Nigerian population. The reported completion rate is greater than the reported average for all the geopolitical zones and the national average ( McGavin *et al.*, 2018). This difference can be attributed to the higher education, understanding of childhood immunization, and employment of the women in this population which is higher than the national averages. Also supporting the reports of Hailu *et al.* (2019); Mugada *et al.* (2017) among others, our study revealed the factors contributing to incomplete immunization rate among mothers, which are the cost of special vaccines, such as Rotavirus, mothers’ forgetfulness of immunization schedule and period, distance to immunization centers and seldom shortage of vaccines.

These women, who mostly work in the informal economy, do so without social benefits like pension, health insurance, or sick leave, rather, they seek micro loans ( Omobowale *et al.*, 2020) or develop an *ajǫ (a daily/ weekly/ or monthly contributory saving system)* system ( Omobowale, 2011) to boost their business. Amidst these various survival systems, the informal working mothers must also take cognizance of the health of their children by taking them for immunization, when necessary. Thus, the contextual understanding of these sets of women is germane to the immunization exercise and how such exercise is conceived and practiced. Through the various narratives above, the working mothers, in spite of their various businesses, understand the importance of childhood immunization in the reflection that Immunization is a cost-effective public health policy designed to prevent millions of children from morbidity and mortality ( Ahmad *et al.*, 2017). Childhood immunization is understandably perceived as “prevention” from common childhood diseases such as polio, meningitis, hepatitis, and death, thus, it must be taken seriously. Rather than an exercise, childhood immunization is a culture, which should be preserved through the socialization process and passed down from generation to generation. With such a preservation culture, it is mandatory for all mothers to immunize their child(ren).

The contextual understanding of childhood immunization provides detailed information, as well as a broad analysis of how working mothers conceive the immunization process; what immunization is, and how immunization should be done. This is deeper than the awareness and perceived knowledge of mothers on immunization that are mostly examined by scholars. From working a mother’s perspective, the study gives a clearer picture of what immunization means to them [working mothers]. This simply means that it is not enough for working mothers to be aware of and have a knowledge of childhood immunization, which could merely be through information, but how immunization works and functions. In this case, immunization becomes a prosocial action ( Korn *et al.*, 2020) that benefits working mothers and their children, on the one hand and society, on the other hand. As revealed, although most the working mothers are aware of the functions of childhood immunization, they are ill-informed of the specific functions of the vaccines given to their children. Most working mothers who immunize their child(ren) do so as a result of the general assumption that immunization cures diseases and prevents children from death. The study also shows how mothers, due to a lack of understanding, use supplements to aid the vaccinations received by their children, which could be detrimental to the health of the children Thus, what is lacking is the contextual understanding and interpretation of the functions of the specific vaccines that children receive and the reaction that such vaccines could cause. It is therefore important to also contextualize vaccine names in the indigenous words or phrases as to drive how to establish an in-depth meaning and function of each vaccine.

Conclusively, working mothers’ awareness and knowledge about childhood immunization is not contextualize in practice thus leading to incomplete understanding and interpretation of vaccine and vaccination. Similarly, no mention of the strengths and limitations of your work in this manuscript. This study contribute to the discourse on childhood immunization by identifying socio-cultural, socio-economic and day-to-day taken for grantend determinants that affect post-day one immunization among the study population, however, the study is limited in that the sample cannot be generalised for the whole of Nigeria or West Africa and the data that were collected represent western part of Nigeria. The study recommends further National studies. The study recommends further National studies. Furthermore, understanding the duration/timing of immunization and period plays a significant role in the completion of immunization. As shown, many of the mothers gave different responses in the timing and duration of immunization. Although the majority of the mothers agree that it starts from birth, the ending period for the immunization, however, is yet unknown. The understanding of some mothers is that it ends at 9 months, some say 1 year, yet another set of mothers believe that is 1 year and 6 months and some agreed to 2 years. Of course, variations in the timing and period of immunization will affect the completion rate of immunization, particularly, for working mothers who are striving to make ends meet. In addition, some of the mothers do not know the schedule of each vaccine. Thus, understanding the function of each vaccine and the schedule will invariably help in the successful completion of immunization. This study is however, limited in that it was conducted among mothers in a specifically in South west geographical location of Nigeria and may not be generalized for the country.

## Data Availability

Due to ethical constrains to protect the privacy of the participants, the raw qualitative data containing identifiable information has not been made publicly available. However, access to the data can be made under specific documented request that must confirm that the data will not be made public or misused through the corresponding author. The underlying quantitative data can be found below: Harvard Dataverse: "Standard baseline result for working mothers in Ibadan SHEVACCS", https://doi.org/10.7910/DVN/EHQBQL ( Omobowale, 2024). Olukemi Amodu, Mofeyisara Omobowale, Folakemi Amodu, 2024, "Replication Data for: Shevaccs", https://doi.org/10.7910/DVN/D9LADS, Harvard Dataverse, V2, UNF:6:xnmsTYuwFyYysVObLcAYww== [fileUNF] Harvard Dataverse: "Standard baseline result for working mothers in Ibadan SHEVACCS", https://doi.org/10.7910/DVN/EHQBQL ( Omobowale, 2024). This project contains the following extended data: consent for informal mothers.docx participants general info page sample.docx Data are available under the terms of the Creative Commons Zero "No rights reserved" data waiver (CC0 1.0 Public domain dedication).

## References

[ref-1] Abbas K Procter SR van Zandvoort K : Routine childhood immunisation during the COVID-19 pandemic in Africa: a benefit-risk analysis of health benefits versus excess risk of SARS-CoV-2 infection. *Lancet Glob Health.* 2020;8(10):e1264–72. 10.1016/S2214-109X(20)30308-9 32687792 PMC7367673

[ref-2] Abdullahi S : Factors affecting completion of childhood immunization in north west Nigeria. * World J Vaccines.* 2018;4(4):175–183. Reference Source

[ref-3] Abdulraheem IS Onajole AT Jimoh AAG : Reasons for incomplete vaccination and factors for missed opportunities among rural Nigerian children. *J Public Health Epidemiol.* 2011;3(4):194–203. Reference Source

[ref-4] Adedokun ST Uthman OA Adekanmbi VT : Incomplete childhood immunization in Nigeria: a multilevel analysis of individual and contextual factors. *BMC Public Health.* 2017;17(1): 236. 10.1186/s12889-017-4137-7 28270125 PMC5341359

[ref-5] Adeloye D Jacobs W Amuta AO : Coverage and determinants of childhood immunization in Nigeria: a systematic review and meta-analysis. *Vaccine.* 2017;35(22):2871–2881. 10.1016/j.vaccine.2017.04.034 28438406

[ref-6] Adenike OB Adejumoke J Olufunmi O : Maternal characteristics and immunization status of children in north central of Nigeria. * Pan Afr Med J.* 2017;26:159. 10.11604/pamj.2017.26.159.11530 28588745 PMC5446779

[ref-8] Adeyinka DA : Uptake of childhood immunization among mothers of under-five in southwestern Nigeria. *The Internet Journal of Epidemiology.* 2012;7(2).

[ref-10] Ahmad NA Jahis R Kuay LK : Primary immunization among children in Malaysia: reasons for incomplete vaccination. *J Vaccines Vaccin.* 2017;8:358. 10.4172/2157-7560.1000358

[ref-42] Alabi MA Fasasi MI Obiora RU : Factors associated with full childhood vaccination coverage among young mothers in Northern Nigeria. *Pan Afr Med J.* 2024;47: 4. 10.11604/pamj.2024.47.4.37517 38371647 PMC10870161

[ref-11] Antai D : Migration and child immunization in Nigeria: individual- and community-level contexts. *BMC Public Health.* 2010;10: 116. 10.1186/1471-2458-10-116 20211034 PMC2847974

[ref-12] Antai D : Gender inequities, relationship power, and childhood immunization uptake in Nigeria: a population-based cross-sectional study. * Int J Infect Dis.* 2012;16(2):e136–e145. 10.1016/j.ijid.2011.11.004 22197748

[ref-14] Anyene BC : Routine immunization in Nigeria: the role of politics, religion and cultural practices. *African Journal of Health Economics.* 2014;03(1):01–09. Reference Source

[ref-51] Asim M Mahmood B Sohail MM : Infant health care practices in Pakistan: a systematic review. *The Professional Medical Journal.* 2015;22(08):978–988. 10.29309/TPMJ/2015.22.08.1142

[ref-13] Brounéus K : In-depth interviewing. *Understanding Peace Reasearch: Methods and Challenges.* 2011;130–45. Reference Source

[ref-15] Chido-Amajuoyi OG Wonodi C Mantey D : Prevalence and correlates of never vaccinated Nigerian children, aged 1–5 years. *Vaccine.* 2018;36(46):6953–6960. 10.1016/j.vaccine.2018.10.006 30337173

[ref-16] Ekwebelem OC Nnorom-Dike OV Aborode AT : Eradication of wild poliovirus in Nigeria: lessons learnt. * Public Health Pract (Oxf).* 2021;2: 100144. 10.1016/j.puhip.2021.100144 36101607 PMC9461633

[ref-17] Fatiregun AA Okoro AO : Maternal determinants of complete child immunization among children aged 12–23 months in a southern district of Nigeria. *Vaccine.* 2012;30(4):730–736. 10.1016/j.vaccine.2011.11.082 22137878

[ref-18] Hailu S Astatkie A Johansson KA : Low immunization coverage in Wonago district, southern Ethiopia: a community-based cross-sectional study. *PLoS One.* 2019;14(7): e0220144. 10.1371/journal.pone.0220144 31339939 PMC6655723

[ref-19] Hilton S Petticrew M Hunt K : ‘Combined vaccines are like a sudden onslaught to the body’s immune system’: parental concerns about vaccine ‘overload’ and ‘immune-vulnerability’. *Vaccine.* 2006;24(20):4321–7. 10.1016/j.vaccine.2006.03.003 16581162

[ref-20] Ijarotimi IT Fatireun AA Adebiyi OA : Urban-rural differences in immunisation status and associated demographic factors among children 12–59 months in a southwestern state, Nigeria. *PLoS One.* 2018;13(11): e0206086. 10.1371/journal.pone.0206086 30395617 PMC6218029

[ref-21] Korn L Böhm R Meierf NW : Vaccination as a social contract. *Proc Natl Acad Sci U S A.* 2020;117(26):14890–14899. 10.1073/pnas.1919666117 32541033 PMC7334515

[ref-22] McGavin ZA Wagner AL Carlson BF : Childhood full and under-vaccination in Nigeria, 2013. *Vaccine.* 2018;36(48):7294–7299. 10.1016/j.vaccine.2018.10.043 30340882

[ref-23] Miles MB Huberman AM : Qualitative data analysis: an expanded sourcebook.sage,1994. Reference Source

[ref-24] Mugada V Chandrabhotla S Kaja DS : Knowledge towards childhood immunization among mothers & reasons for incomplete immunization. *J App Pharm Sci.* 2017;7(10):157–161. 10.7324/JAPS.2017.71023

[ref-25] Mutiu A Yahaya A Baban B : Immunization, primary healthcare system and efficient service delivery in Nigeria. *International Journal of Recent Innovations in Academic Research.* 2019;3(12):11–38. Reference Source

[ref-26] National Population Commission (NPC) Nigeria and ICF Macro: Nigerian Demographic and Health Survey (NDHS) 2013. 2014. Reference Source

[ref-41] Nigeria Population Commission: Nigeria demographic and health survey 2018. NPC, ICF,2018. Reference Source

[ref-27] Obiajunwa PO Olaogun AA : Childhood immunization coverage in south west Nigeria. *Sudanese J Public Health.* 2013;8(3):94–98. Reference Source

[ref-28] Oladepo O Dipeolu IO Oladunni O : Nigerian rural mothers’ knowledge of routine childhood immunizations and attitudes about use of reminder text messages for promoting timely completion. *J Public Health Policy.* 2019;40(4):459–477. 10.1057/s41271-019-00180-7 31427672 PMC7771534

[ref-30] Oladokun RE Adedokun BO Lawoyin TO : Children not receiving adequate immunization in Ibadan, Nigeria: what reasons and beliefs do their mothers have? *Niger J Clin Pract.* 2010;13(2):173–8. 20499751

[ref-29] Oladokun RE Lawoyin TO Adedokun BO : Immunization status and its determinants among children of female traders in Ibadan, south-western Nigeria. *Afr J Med Med Sci.* 2009;38(1):9–15. 19722422

[ref-31] Oleribe O Kumar V Awosika-Olumo A : Individual and socioeconomic factors associated with childhood immunization coverage in Nigeria. *Pan Afr Med J.* 2017;26: 220. 10.11604/pamj.2017.26.220.11453 28690734 PMC5491752

[ref-33] Omobowale AO : Social capital and *AJỌ* system among working class traders in Ibadan, Nigeria. *J Labor Soc.* 2011;14(3):333–346. 10.1111/j.1743-4580.2011.00344.x

[ref-34] Omobowale MO : Class, gender, sexuality, and leadership in Bodija market, Ibadan, Nigeria. *J Anthropol Res.* 2019;75(2):235–251. 10.1086/702708

[ref-35] Omobowale MO : "You will not mourn your children": spirituality and child health in Ibadan urban markets. *J Relig Health.* 2021;60(1):406–419. 10.1007/s10943-020-01032-5 32436036

[ref-36] Omobowale MO : Standard baseline result for working mothers in Ibadan SHEVACCS. Harvard Dataverse, V1. [Dataset].2024. 10.7910/DVN/EHQBQL

[ref-32] Omobowale AO Oyelade OK Omobowale MO : Contextual reflections on COVID-19 and informal workers in Nigeria. *Int J Sociol Soc Policy.* 2020;40(9/10):1041–1057. 10.1108/IJSSP-05-2020-0150

[ref-37] Ophori EA Tula MY Azih AV : Current trends of immunization in Nigeria: prospect and challenges. *Trop Med Health.* 2014;42(2):67–75. 10.2149/tmh.2013-13 25237283 PMC4139536

[ref-38] Tade O : ‘My husband is living like a dead person’: explaining women portage labour in Ibadan urban market. *African Identities.* 2022;20(3):225–236. 10.1080/14725843.2020.1813550

[ref-39] Tagbo BN Uleanya ND Nwokoye IC : Mothers’ knowledge, perception and practice of childhood immunization in Enugu. *Niger J Paed.* 2012;39(3):90–96. 10.4314/njp.v39i3.1

[ref-40] World Health Organization Africa (WHO): Seventy seven percent (77%) of children 12 – 23 months in Nigeria did not receive all routine immunization – Survey findings. 2017. Reference Source

