# Supplementary material for: Contextualizing post day-one childhood immunization in-take drop-off rate in Nigeria: An assessment of working mothers in Ibadan
Source: Gates Open Res. 2025 May 27;8:48. Originally published 2024 May 31. [Version 2] doi: 10.12688/gatesopenres.15135.2 (PMC12287506; doi:10.12688/gatesopenres.15135.2)
Supplement: Peer review history

# Reviewer report: Reviewer response for version 2

**DOI:** 10.21956/gatesopenres.17713.r39706

**Published:** 2025-07-23

**Authors:** Kennedy Oberhiri Obohwemu

**Affiliations:** 1 Oxford Brookes University, Oxford, England, UK

The authors have written a robust, well-conceptualized mixed-methods study that investigates a critical and understudied public health issue—vaccine drop-off rates beyond initial immunization among informal working mothers in urban Nigeria. The paper is theoretically grounded, methodologically sound, and socio-culturally nuanced. Below is a detailed critique evaluating its strengths, limitations, and overall contribution to the field.

**Strengths of the Study**

1. **Rich Contextualization and Relevance**:
  The article is commendable for its sociocultural depth. By focusing specifically on informal working mothers in Ibadan—a population structurally disadvantaged yet often underrepresented in immunization research—it unearths a unique set of barriers to vaccine completion. This focus helps bridge the gap between broad immunization statistics and lived maternal experiences.
2. **Comprehensive Mixed-Methods Design**:
  The use of both quantitative (n=1,044) and qualitative (n=73) methods allows for a triangulated understanding of immunization behavior. Quantitative data offers generalizable trends, while qualitative narratives expose perceptions, misinterpretations, and coping strategies. The study design is particularly strong due to the explanatory sequential approach, where qualitative findings enrich statistical results.
3. **Thematic Depth and Cultural Translation**:
  The research captures how mothers linguistically and conceptually understand vaccines (e.g., polio referred to as “romolapa-romolese”). This enables practical recommendations such as translating medical terms into indigenous languages, a culturally sensitive strategy often lacking in conventional health communication campaigns.
4. **Inclusion of Non-NPI Vaccines**:
  The study’s attention to “special vaccines” like Rotavirus and PCV—often missed due to cost or unawareness—highlights systemic inequities between public health offerings and actual community needs. This issue is particularly relevant in low-income settings where public immunization schedules lag behind WHO-recommended comprehensive coverage.
5. **Policy and Practice Implications**:
  The article recommends actionable solutions, including improved health education, decentralization of services, and financial subsidies for non-NPI vaccines. These recommendations align well with Nigeria's current efforts to improve immunization uptake and are grounded in empirical evidence from the field.

**Limitations and Areas for Improvement**

1. **Limited Generalizability**:
  While the study provides detailed insights into Ibadan’s informal working mothers, the findings may not be representative of all Nigerian or West African contexts. The authors acknowledge this but a clearer delimitation in the abstract and discussion would strengthen interpretive caution.
2. **Inconsistent Presentation of Data**:
  While the data are rich, the structure of tables and figures is not consistently integrated into the discussion. For example, the completion rates for various vaccines are compelling but are sometimes buried in narrative instead of clearly tabulated or visually summarized.
3. **Overextended Qualitative Quotations**:
  The study would benefit from a tighter synthesis of qualitative data. While the inclusion of participant voices is vital, some quotes are unnecessarily long and repetitive. More concise, thematic presentation (e.g., matrix tables with representative quotes) would increase clarity.
4. **Vaccine Knowledge Assessment Lacks Quantification**:
  Although mothers’ knowledge gaps are described vividly, the study could be improved by quantifying these gaps—perhaps via a vaccine knowledge index or score—so that educational deficiencies can be measured and tracked over time.
5. **Absence of Health Worker Perspectives**:
  While the study mentions interviews with health workers, their perspectives are not sufficiently integrated into the analysis. Understanding their constraints (e.g., time pressures, vaccine stock-outs) would complement the maternal narratives and provide a more complete view of the service delivery ecosystem.
6. **Lack of Analytical Rigor on Causal Factors**:
  Although chi-square and t-tests were used, multivariate logistic regression could have offered more nuanced insights into which variables most strongly predict immunization drop-off. Without this, the interpretation of associations remains largely descriptive.
7. **No Formal Strengths and Limitations Section**:
  The study’s discussion informally mentions limitations, but it lacks a clearly delineated section summarizing the methodological constraints and their potential impact. Formalizing this would align the paper more closely with standard reporting guidelines like STROBE or COREQ.

Is the work clearly and accurately presented and does it cite the current literature?

Yes

If applicable, is the statistical analysis and its interpretation appropriate?

Yes

Are all the source data underlying the results available to ensure full reproducibility?

No

Is the study design appropriate and is the work technically sound?

Yes

Are the conclusions drawn adequately supported by the results?

Yes

Are sufficient details of methods and analysis provided to allow replication by others?

Yes

Reviewer Expertise:

Epidemiology of infectious and non-infectious diseases, Vaccinations, Public health promotion

I confirm that I have read this submission and believe that I have an appropriate level of expertise to confirm that it is of an acceptable scientific standard.

# Reviewer report: Reviewer response for version 1

**DOI:** 10.21956/gatesopenres.16473.r37382

**Published:** 2024-09-03

**Authors:** Monica Jain

**Affiliations:** 1 International Initiative for Impact Evaluation, Washington, District of Columbia, USA

The study has an important objective of understanding of informal working mothers of the concept of immunization, vaccines not under the National Program on Immunization (NPI), immunization schedule and period as well as the timing and duration of the immunization. My main comments are as follows:

1. The paper needs major editing as there is a significant repetition in different sections of the papers and correction in errors leave some sentences hanging and make it difficult to follow authors’ reasoning.
2. Methods - Which software was used for doing the process coding and content analysis?
3. Methods – While authors mention that that markets were randomly selected, they do not mention the criteria of selection of women within the markets.
4. The authors mention – ‘’However, in all the markets, market leaders and significant others of the mothers had earlier been identified, visited, and carried along from the inception of the study.’’ What is the role of the market leaders and significant others in the study design, data and analysis?
5. Statistical analysis – In table 3 – What is general population being referred to? Is it an average of urban and rural population?
6. Statistical analysis - In figure 1 – the number of PCV or OPV vaccination is not visible.
7. It would be useful to provide a rationale for understanding the uptake of ‘’special vaccines’’ not under NPI? Also, what vaccines are under this ‘’special vaccines’’ category?
8. In one part of the paper authors mention ‘’the factors contributing to incomplete immunization rate among mothers, which are the cost of special vaccines, such as Rotavirus.’’ If the vaccines are not part of the national immunization schedule, should they be included in estimation of immunization completion rate? Are these vaccines used in calculating childhood immunization completion rate and drop-off rate in Table 3? If not, then it may be useful to differentiate between the immunization completion rates under the NPI schedule and those with special vaccines.
9. Can the authors shed more light on the ‘’supplements’’ that they have mentioned in the paper? Why are these supplements important to discuss in relation to immunization uptake?
10. The results based on qualitative data contain too many similar quotes. Removing similar quotes will improve readability of the paper.
11. Authors mention – ‘’In some cases, many of them despite being married, still remained “breadwinners” in their families’’. It is not clear what the authors are suggesting about roles of women and their work status and after marriage.
12. Authors mention – ‘’Rather than an exercise, childhood immunization is a culture, which should be preserved through the  socialization  process and passed down from generation to generation. With such a preservation culture, it is mandatory for all mothers to immunize their child(ren).’’ Are the authors implying that childhood immunization is embedded in the culture so much so that it is mandatory for mothers to immunize their children? Please clarify the argument being made here and its implication.

Is the work clearly and accurately presented and does it cite the current literature?

Partly

If applicable, is the statistical analysis and its interpretation appropriate?

Yes

Are all the source data underlying the results available to ensure full reproducibility?

Yes

Is the study design appropriate and is the work technically sound?

Yes

Are the conclusions drawn adequately supported by the results?

Partly

Are sufficient details of methods and analysis provided to allow replication by others?

Partly

Reviewer Expertise:

Public health policy with specialization in immunization of children.

I confirm that I have read this submission and believe that I have an appropriate level of expertise to confirm that it is of an acceptable scientific standard, however I have significant reservations, as outlined above.

# Reviewer report: Reviewer response for version 1

**DOI:** 10.21956/gatesopenres.16473.r37387

**Published:** 2024-07-22

**Authors:** Olayinka Ibrahim

**Affiliations:** 1 University of Ilorin Teaching Hospital,, Ilorin, Nigeria; 2 Pediatrics, University of Global Health Equity, Kigali, Kigali, Rwanda

Re: ﻿Contextualizing post day-one childhood immunization in-take

 drop-off rate in Nigeria: An assessment of working mothers in

 Ibadan

 Comments

 Title “﻿Contextualizing post day-one childhood immunization in-take

 drop-off rate in Nigeria: An assessment of working mothers in

 Ibadan”  This title is too technical for general audience and a bit confusing, what is post day-one childhood immunization? Is after birth? Is first dose irrespective of date vaccine is received? The phrase is unclear? This should be modified. Immunization is an important, and audience go beyond medical personnel's. Also, be specific. add informal working mother: Typical understanding of a working mothers in Nigeria refers to those in formal sector, and also, include country? Not everyone knows Ibadan Globally

 Abstract

 Aims: “﻿perceive post-day-one routine immunization” –this manuscript did not access post one day routine immunization but rather mothers’ perception and knowledge of immunization along with other itemized aims.

 Methods

 “﻿ working nursing mothers” qualify it further: informal working nursing mothers.

 Results:  Average age (mean) should be presented along the standard deviation. “﻿The mean age of mothers at their first childbirth within the study group was 24.12 years” delete, it is not adding any information

 Results

 ﻿A “significant majority” of these mothers 95%) are married—delete significant

 “﻿ livelihood related causes” include the related causes? Though I find it difficult to locate in the manuscript

 Conclusion

 “﻿ It is crucial for mothers to possess comprehensive knowledge about the mechanics and

 operation of immunization” ?difficult to discern this the manuscript

 Introduction

 Unduly long with most concept repeated; this should be reduce and preferably about half of the current number of pages.

 Methods

 When was this data collected???

 “﻿A total of 1,044 (quantitative samples)” ? How is this sample size derived? Provide the details,

 The qualitative sample is adequate.

 “﻿Immunization record assessment” how was this done? Provide the details.

 “﻿Health workers in the selected study sites” were they part of the qualitative vs quantitative? I did see their findings in the results.

 What informed the sampling of 13 markets? How were the1,044 mothers recruited across these markets?

 “﻿The dataset was retrieved and analyzed using statistical packages for Social Sciences (SPSS, version 20 windows). Data were represented by numbers, percentages and expressed by mean. Chi-square test was used to observe the difference between the proportions, t-test was applied to observe- the difference between the two means for normally distributed data”

 I guess this was the quantitative arm of the study, kindly specify

 Also, this too  generic, give details of how each variable was handled. Indeed, there was no a single inferential statistics?

 Results

 All the mean should be accompanied with their respective standard deviations

 “﻿The mean age of mothers enrolled in this study was 31.39 years. The mean age of mothers at first childbirth in the study population is 24.12 years” difficult to understand while the mean age of the mother and mean age at first childbirth? Look more of tautology??? The most important- age of the children of the mothers’ recruited is missing, which will guide in the interpretation of the data?

 “﻿The majority of mothers (53.8%)” In science 53% is not majority? Rather report as about half of the mothers

 “﻿The majority of the mothers (95%) in this study were married while only 43 (4.2%) were single. The majority of mothers (82.9%) that had antenatal care during their pregnancy attended health care facilities for their antenatal services, while 13.6% had their antenatal care in faith homes. About 76% and 16.4% of respondents in this population delivered their last child at a healthcare facility and mission house, respectively” These information are incomplete and yet there is no reference Table??? Besides, most of the information was not subjected to statistical analysis? What is there relevance

 “﻿A higher number of mothers in the urban markets completed the immunization

 schedule for their children till the 14th week (42.57%) and the ninth month (33.95%)”- you can not use completion rate rate for 14 ^th^ week because it is not the end point, similar observation for 9 months- kindly revise and use drop off rate.

 “﻿Completion rates in Table 3 were visibly lower among children of mothers in the rural market. More than half (53.3%) of the children of mothers in the rural markets dropped off the immunization schedule before they were fully vaccinated” you cannot make this conclusion without inferential statistics,-cursory look is insufficient.

 Table 1: provide the details of “others” as footnote

 “﻿Table showing the highest educational attainment by mothers in the study. This

 table shows 37 of the mothers do not have any form of education, the majority (536)

 completed secondary school education and 180, making about 18% had tertiary

 education degrees” Delete…you can not have a prose repetition of a Table

  “About 48 mothers” humans are countable noun and can not be about?

 Table 2: Present column one in days for consistency and need for clarity does within first week include day zero, does within second week include preceding days

 Table 3: Insert n? percent's alone does not convey the true meanings? Delete completion rate, you can specify drop rate at 14 weeks, at 9 months? Nothing like completion rate- it gives impression as if it is satisfactory . Delete the footnote, besides it was a wrong interpretation.

 Figure 1: Again the completion rate is confusing? This figure should n somewhere?

 “﻿Post-day-one childhood immunization is an exercise that should be taken seriously. In explaining some of the diseases that post-day-one immunization prevents” ? birth dose vaccines are equally important (hepatitis B, Polio and BCG?

 “﻿However, in spite of the working mothers’ enlightenment on childhood immunization, some of the mothers do not know the specific vaccines their children receive. Many of them take the vaccines as instructed and on the general assumption that ‘it is good for children’ as adduced by one of the interviewees” It would have been good to also find out why don’t know the names? Are their local names for these vaccines?

 “﻿supplements’ this term needs to be defined? Supplement may mean different things to different people.

 ﻿Also, I used to hear the advertisement, because they don’t write the name of the vaccine for us, they just write 3 letters, and you know it is only the nurses who know what they mean by that” Partly it is not their faulty, if they were not told ? efforts should be made to teach the mothers the corresponding local names for these vaccines if there is any?

 ﻿iko ahubi (coarse cough)? Am not sure the correct interpretation/translation is coarse cough -rather cough with post-tussive vomiting/pertussis),

 “﻿Of all the vaccines, the BCG is very important, the PCV, and the Rotavirus. They are all good. For the Rotavirus, if not that it is expensive for most of the parents, it would have been good for everybody to take it for their children (Gbagi IDI)” am not sure when this data was collected but you should discuss with current NPI schedules, Rota Virus is now part of our routine immunization and PCV has already been part of pentavalent.

 “﻿Rotavirus is very low as shown that only a 7.5% rate had been completed (Table 2).? I can not see this in Table 2???

 “ Mothers/caregivers’ knowledge of routine childhood immunization and vaccination status in children aged, 12-23 months in Ilorin, Nigeria; SO Ariyibi, AI Ojuawo, RM Ibraheem, FM Afolayan, OR Ibrahim African Health Sciences 23 (4), 582-591

 Above article may be relevant for your discussion.

Is the work clearly and accurately presented and does it cite the current literature?

Partly

If applicable, is the statistical analysis and its interpretation appropriate?

No

Are all the source data underlying the results available to ensure full reproducibility?

Partly

Is the study design appropriate and is the work technically sound?

Partly

Are the conclusions drawn adequately supported by the results?

Partly

Are sufficient details of methods and analysis provided to allow replication by others?

Partly

Reviewer Expertise:

Pediatric Infectious disease, General Pediatrics

I confirm that I have read this submission and believe that I have an appropriate level of expertise to confirm that it is of an acceptable scientific standard, however I have significant reservations, as outlined above.

## References

[1] : Mothers/caregivers' knowledge of routine childhood immunization and vaccination status in children aged, 12-23 months in Ilorin, Nigeria. *Afr Health Sci* .2023;23(4) : 10.4314/ahs.v23i4.61 582-591 10.4314/ahs.v23i4.61 38974273 PMC11225479

# Response

**Published:** 2024-09-04

**Authors:** mofeyisara omobowale

**Affiliations:** Institute of Child Health, University of Ibadan, Ibadan, Oyo, Nigeria

Dear Reviewer,

 Thank you for your insightful review. All suggestions have been examined and used in the improvement of the manuscript.

# Reviewer report: Reviewer response for version 1

**DOI:** 10.21956/gatesopenres.16473.r37252

**Published:** 2024-07-17

**Authors:** Charles Olomofe

**Affiliations:** 1 East Tennessee State University, Johnson City, Tennessee, USA

I have a few comments on the research.

 METHODS SECTION

 Every research carries risk. I would rather say the risk is minimal. What about the data that was collected? If it gets into the wrong hands, that is a risk!

 Conducting an interviewer-administered questionnaire for 1,044 persons is huge. Did you make use of research assistants? If yes, you need to mention it here.

 Moreover, which language was this questionnaire administered? Was Yoruba language employed at any point? If yes, was a back translations done?

 Can you expound on this (sequential mixed method) more? Any sampling technique used?

 Any particular reason or justification why only women within markets were considered? Working mothers in the title of your research is a broad group and it would be great to know why you scoped only market women.

 DISCUSSION SECTION

 There is no conclusion in this manuscript. What important messages are you leaving your readers with?

 Similarly, no mention of the strengths and limitations of your work in this manuscript. Are there things you would have done differently had it been the circumstances were different

Is the work clearly and accurately presented and does it cite the current literature?

Yes

If applicable, is the statistical analysis and its interpretation appropriate?

Yes

Are all the source data underlying the results available to ensure full reproducibility?

Yes

Is the study design appropriate and is the work technically sound?

Yes

Are the conclusions drawn adequately supported by the results?

Partly

Are sufficient details of methods and analysis provided to allow replication by others?

Partly

Reviewer Expertise:

Infectious disease epidemiology and vaccine research

I confirm that I have read this submission and believe that I have an appropriate level of expertise to confirm that it is of an acceptable scientific standard, however I have significant reservations, as outlined above.

# Response

**Published:** 2024-09-04

**Authors:** mofeyisara omobowale

**Affiliations:** Institute of Child Health, University of Ibadan, Ibadan, Oyo, Nigeria

**Reviewer comment:** 1. Every research carries risk. I would rather say the risk is minimal. What about the data that was collected? If it gets into the wrong hands, that is a risk!:****

**Author response: **The participants in this study were not exposed to any serious know risk. The research team employed various means of protection to minimize risk.

**Reviewer comment: **2. Conducting an interviewer-administered questionnaire for 1,044 persons is huge. Did you make use of research assistants? If yes, you need to mention it here: 

**Author response: ** Yes. Trained research assistants were employed on the project to assist in data collection. 

**Reviewer comment: **3. Can you expound on this (sequential mixed method) more?: Sequential mixed method here implies timing in collection of the data. The phasing of how data were collected. Any sampling technique used?: 

**Author response: **Explanatory Sequential Design detailing two phase design,  where quantitative data is collected and analyzed first, then qualitative data were also collected and analyzed based on the quantitative results.

**Reviewer comment: **4. Any particular reason or justification why only women within markets were considered? Working mothers in the title of your research is a broad group and it would be great to know why you scoped only market women:

**Author response:** Working mothers have limited or no time to attend the available immunization centers because of the time-consuming age-long practices of traditional immunization centers, which can result in loss of time, wages and in some instances source of livelihood for these mothers. These partly culminate to low demand for childhood vaccination in addition, many working mothers in the informal sectors like the local markets miss out on community household’ immunization programmes due to the long hours they spend in the market and also because of the inconvenience of leaving their wares to attend immunization centers, and there are limited studies in Nigeria on mothers on informal economic space  and childhood immunization in particular, thus this study was carried out.

 DISCUSSION SECTION

**Reviewer comment: **5. There is no conclusion in this manuscript. What important messages are you leaving your readers with?:

**Author response: ** Conclusively, working mothers’ awareness and knowledge about childhood immunization is not contextualize in practice thus leading to incomplete understanding and interpretation of vaccine and vaccination.

**Reviewer comment: **6.Similarly, no mention of the strengths and limitations of your work in this manuscript. Are there things you would have done differently had it been the circumstances were different:  

**Author response: **This study contribute to the discourse on childhood immunization by identifying socio-cultural, socio-economic and day-to-day taken for granted  determinants that affect post-day one immunization among the study population, however, the study is limited in that the sample cannot be generalized for the whole of Nigeria or West Africa and the data that were collected represent western part of Nigeria. The study recommends further National studies.
